# Frontal Theta Event‐Related Oscillations During a Continuous Performance Test: The Influence of Trauma Type and Fluid Intelligence Polygenic Score

**DOI:** 10.1002/brb3.70729

**Published:** 2025-08-04

**Authors:** Stacey Saenz de Viteri, Ashwini Pandey, Chella Kamarajan, Gayathri Pandey, Weipeng Kuang, Sivan Kinreich, Grace Chan, Martin H. Plawecki, Marc A. Schuckit, Bernice Porjesz, Jacquelyn L. Meyers

**Affiliations:** ^1^ Department of Psychiatry State University of New York Downstate Health Sciences University Brooklyn New York USA; ^2^ Department of Psychiatry University of Connecticut School of Medicine Farmington Connecticut USA; ^3^ Department of Psychiatry Indiana University Indianapolis Indiana USA; ^4^ Department of Psychiatry University of California San Diego Medical School San Diego California USA

## Abstract

**Background:**

Trauma exposure during adolescence can lead to impaired executive function and altered neural development in related cognitive control networks. Studies have shown that adolescents with a family history of alcohol use disorders have a disproportionately high rate of trauma exposure, as well as impaired response inhibition, making them particularly vulnerable to cognitive impairment and poor mental health outcomes in adulthood. While studies have suggested that this may be due partly to genetic influences, no study to our knowledge has investigated the influences of trauma exposure and polygenic scores (PGS) for cognitive function on later cognitive function.

**Methods:**

This study used data from trauma‐exposed individuals in the Collaborative Study on the Genetics of Alcoholism prospective cohort (*N* = 912), comprising offspring from alcohol‐dependent high‐risk and comparison families, to investigate main and interaction effects of PGS for cognitive function (fluid intelligence score, UK Biobank study) and trauma exposure (nonsexual assaultive, nonassaultive, sexual assaultive) on performance measures and frontal theta event‐related oscillations (EROs) during a continuous performance test (CPT).

**Results:**

A significant interaction between fluid intelligence PGS and nonsexual assaultive trauma was observed for CPT ERO power (*B* = 0.094, *p *< 0.01), such that individuals with a lower PGS who experienced a nonsexual assaultive traumatic exposure had lower frontal theta ERO power during the cued no‐go condition of the CPT.

**Conclusion:**

These findings suggest that a polygenic predisposition for higher fluid intelligence may be associated with differences in neural response inhibition depending on trauma type.

## Graphical abstract

Individuals with lower fluid intelligence polygenic scores and exposure to a nonsexual assaultive trauma had lower frontal theta event‐related oscillation power during the cued no‐go condition of the continuous performance test, indicating less efficient neural synchronization during behavioral inhibition.

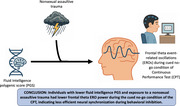

## 1 Introduction

It is estimated that 50%–89% of the United States population is exposed to some type of trauma, such as physical or sexual assault, or witnessing violence or a natural disaster (Kilpatrick et al. 2013; Knipscheer et al. 2020). Trauma exposure can increase the risk of mental health problems, such as post‐traumatic stress disorder (PTSD) and substance use disorders (SUDs) (Breslau 2009; Keyes, Hatzenbuehler, and Hasin 2011). Severity varies by trauma type, such as nonsexual assaultive, nonassaultive, and sexual assaultive trauma (Ozer et al. 2003). Individuals exposed to assaultive trauma, in particular, are more likely to develop PTSD (Cisler et al. 2011), highlighting the importance of trauma type in risk for mental health problems.

Trauma exposure during critical developmental periods, such as adolescence, is associated with worse mental health and impaired executive functions than in adulthood (Dube et al. 2001; Follette, Polusny, Bechtle, and Naugle 1996; Green et al. 2000; Khoury et al. 2010). During adolescence, one specific executive function, response inhibition, is particularly undeveloped and associated with increased risk‐taking behaviors, such as experimentation with substances (Romer Thomsen et al. 2018). A study of individuals with bipolar disorder compared to unaffected individuals with or without a history of childhood trauma showed greater dysfunction in inhibitory control in individuals with a history of childhood trauma compared to those without childhood trauma, regardless of a bipolar disorder diagnosis (Marshall et al. 2016). Trauma, particularly assaultive trauma, during adolescence is associated with altered response inhibition (Bounoua, Miglin, Spielberg, and Sadeh 2020), suggesting trauma type plays a significant role in these associations. Studies have also shown that deficient response inhibition is correlated with trauma‐related disorders, including PTSD and SUDs (Gilpin and Weiner 2017). However, few studies investigate these associations, especially by trauma type, in high‐risk samples.

Electrophysiological measures measured from electroencephalogram during cognitive and behavioral tasks, like event‐related potentials (ERPs) and event‐related oscillations (EROs), are used to investigate brain function during tasks in trauma‐exposed individuals (Lobo et al. 2015). ERP components (e.g. P3(00)) provide time‐domain measures that have been useful as biomarkers for psychopathology. However, P3 is not a unitary phenomenon, but an amalgam of different frequency bands emanating from different brain regions, representing several complex cognitive processes involved in P3. Time‐frequency ERO measures tease apart ERP P3 into an earlier frontal theta (3.0–7.5 Hz, 200–400 ms) and later posterior delta (1.0–3.5 Hz, 300–700 ms), which serve different aspects of complex cognitive processing in P3, providing more specific measures of aspects of brain function (Porjesz and Rangaswamy 2007). Frontal theta has been associated with executive functions, such as response inhibition, and frontal theta EROs during response inhibition have been used as biological markers to identify and understand neurocognitive mechanisms of mental health disorders, such as alcohol use disorders (AUDs) (Kamarajan et al. 2006). Kamarajan et al. (2006) showed lower ERO power in the no‐go condition of a go/no‐go task, first observed in individuals with AUD, was also seen in offspring from families at high risk for AUDs, suggesting neural deficits may precede the development of psychiatric disorders (Kamarajan et al. 2006). Adolescents with a family history of AUD have disproportionately high rates of trauma exposure (Breslau et al. 2013; Dube et al. 2001). Individuals exposed to sexual assaultive trauma also show lower frontal theta EROs during the no‐go condition in a response inhibition task, even after controlling for other factors, including parental AUD (Meyers et al. 2019), suggesting trauma type plays a critical role in ERO differences.

One explanation for similar EROs in individuals with AUD and their offspring may be genetic risk for altered neurocognitive function. Recent studies have conducted genome‐wide association studies (GWAS) on cognitive functions like fluid intelligence (Watanabe et al. 2019) and behavioral measures during cognitive tasks (Davies et al. 2016). Polygenic scores (PGS) for cognition, derived from GWAS on general intelligence and education, have been shown to predict mental health disorders like schizophrenia, (Richards et al. 2020) and cognitive decline (Ding et al. 2019). Gene x environment studies show genetic variants moderate associations between trauma and neuropsychiatric traits like AUD and PTSD (Koenen et al. 2009), but no study has examined how trauma moderates the relationship between cognitive function PGS and neural response inhibition. Since trauma‐related deficits in neural response inhibition have been shown to increase risk for AUD and PTSD, considering genetic vulnerability to complex cognitive functions via polygenic scores will bridge this gap in knowledge and inform future research on risk and resilience pathways.

The current study aimed to investigate interaction effects of trauma type (nonsexual assaultive, nonassaultive, sexual assaultive) and PGS of cognitive function (fluid intelligence) on performance measures and frontal theta ERO power (during cued no‐go and go conditions) from a continuous performance test (CPT), which is used to assess behavioral inhibition, using data from trauma‐exposed offspring in the Collaborative Study on the Genetics of Alcoholism (COGA) prospective study. Exploratory analyses investigate sex differences in these relationships. We hypothesized that assaultive‐type trauma would moderate associations between PGS of cognitive function on performance measures and frontal theta ERO power during a CPT task. Understanding these effects can inform early interventions for individuals at high risk for trauma exposures and altered cognitive function.

## 2 Materials and Methods

### 2.1 Sample and Measures

#### 2.1.1 Sample

This study utilized data from COGA's prospective study, which collected data from 2004–2019. Details on data collection and procedures have been previously published (Bucholz et al. 2017; Dick et al. 2023). COGA's prospective study comprises offspring, aged 12–22 years at enrollment, from alcohol dependent high‐risk and community comparison families. Participants were assessed approximately every 2 years through clinical interview and questionnaires, behavioral, neuropsychological, and neurophysiological assessments. For this cross‐sectional study, data from the last interview were used for each individual who had both neurophysiological and genetic data. Our analyses included participants who were genetically similar to those from European reference panels (i.e., European‐like or European ancestry). We reviewed the National Academies of Sciences, Engineering, and Medicine report on population descriptors and recognize that terminology for genetic ancestry compared to genetic similarity is being considered (NASEM, 2023). Throughout the methods and results, we use the term “European‐like ancestry.”

#### 2.1.2 Continuous Performance Test

A computerized version of the CPT, used to assess response inhibition and adapted from Fallgatter et al. (1998), was administered to individuals from COGA's prospective study (Fallgatter et al. 1998). A total of twelve different letters (A, B, C, D, E, F, G, H, J, L, O, and X) were presented one at a time between two vertical lines as a fixation stimulus. There were 400 trials, where participants were shown the letters “O” and “X” 80 times each, and the other 10 letters 24 times each. Subjects were instructed to press a button when they saw an “X” right after an “O,” but to refrain from pressing the button when they saw an “X” after any other letter. Details regarding ERO processing procedures can be found in Supporting Information.

Performance was measured through total errors (cued go and cued no‐go errors combined; normalized using a square root transformation) and cued Go response time. *S*‐transformed frontal theta (4.0–7.5 Hz, 200–400 ms) total ERO power measured during both cued Go and cued No‐Go were used in this study to measure theta ERO during response activation and inhibition.

#### 2.1.3 Polygenic Scores

PGS for fluid intelligence was generated from summary statistics from the UK Biobank (Watanabe et al. 2019) and was calculated using PRS‐cs (Ge et al. 2019). Data used to calculate PGS were available for 912 trauma‐exposed participants of European‐like ancestry (EA) since this was the only ancestral group used for the UK Biobank GWAS.

#### 2.1.4 Trauma Exposure

Only trauma‐exposed EA individuals (*N* = 912) were included in this study. Trauma exposure was defined using the Semi‐Structured Assessment for the Genetics of Alcoholism (SSAGA‐IV) interview, which includes questions concerning potentially traumatic life events (Bucholz et al. 1994). Three types of traumatic exposures were considered (nonsexual assaultive, nonassaultive, sexual assaultive trauma), which were lifetime binary variables. Nonsexual assaultive trauma included being shot, stabbed, mugged, or threatened with a weapon, kidnapped, or held captive and/or tortured. Nonassaultive trauma included experiencing a life‐threatening accident, experiencing a natural disaster, or unexpectedly discovering a dead body. Sexual assaultive trauma was defined as being raped or sexually assaulted. Descriptive statistics are displayed in Table 1.

**TABLE 1:** Descriptive statistics.

		Trauma exposed (*N* = 912)	Nonsexual assaultive trauma (*N* = 437)	Nonassaultive trauma (*N* = 822)	Sexual assaultive trauma (*N* = 112)
Sex					
	Female	452 (49.6%)	165 (37.8%)	409 (49.8%)	91 (81.3%)
	Male	460 (50.4%)	272 (62.2%)	413 (50.2%)	21 (18.8%)
Age at most recent interview—mean (SD)		21.8 (4.1)	22.4 (4.2)	21.8 (4.1)	23.1 (3.9)
Age at first traumatic exposure (SD)		10.4 (4.9)	14.4 (4.9)	12.3 (5.4)	10.4 (5.5)
Trauma type					
	Nonsexual assaultive	437 (47.9%)	—	347 (42.2%)	66 (58.9%)
	Nonassaultive	822 (90.1%)	347 (79.4%)	—	103 (92.0%)
	Sexual assaultive	112 (12.3%)	66 (15.1%)	103 (12.5%)	—

### 2.2 Statistical Analyses

A linear regression was performed (ESTIMATOR = MLR) in Mplus to test main and interaction effects of trauma type (nonsexual assaultive, nonassaultive, sexual assaultive) and fluid intelligence PGS on CPT performance (total errors, response time to cued Go) and frontal theta ERO measures. Each trauma type was included in the model as mutually exclusive categories and coded as follows: nonsexual assaultive trauma (no = 0, yes = 1), nonassaultive trauma (no = 0, yes = 1), and sexual assaultive trauma (no = 0, yes = 1). The fluid intelligence PGS was treated as a continuous predictor. Covariates included sex (male = 0, female = 1), age, number of CPT sessions, three ancestral principal components, and genotype array. Exploratory analyses were performed to investigate sex differences through a three‐way interaction (PGS × trauma × sex) for any significant interaction from the primary analysis. Prediction values for any significant interactions were calculated and plotted to better understand the nature of these interactions. All measures were included simultaneously in the pathway model to account for correlations observed among variables. Analyses were conducted in *MPlus* (Muthén and Muthén 1998–2017), clustering for familial relatedness (TYPE = COMPLEX). Missing data were handled using full information maximum likelihood. All continuous variables were standardized prior to analysis.

## 3 Results

### 3.1 Main and Interaction Effects of PGS and Trauma on CPT Performance and ERO Measures

Significant main effects for nonsexual assaultive trauma and sexual assaultive trauma such that individuals with a nonsexual assaultive trauma had shorter CPT cued go response times (*B* = −0.089, *p *< 0.05; Table 2) and individuals with a sexual assaultive trauma had more total CPT errors (*B* = 0.092, *p *< 0.05; Table 2) and higher cued no‐go theta ERO power (*B* = 0.085, *p *< 0.05; Table 2). However, no significant main effects remained after a Bonferroni correction for eight tests (*p *< 0.00625; Table 2).

**TABLE 2:** Main and interaction effects of trauma type and neurocognitive polygenic scores (PGS) on continuous performance test (CPT) performance and frontal theta event‐related oscillations (EROs).

	Total errors		Cued go response time		Cued no‐go theta ERO		Cued go theta ERO	
Explanatory Variables	Beta (S.E.)	*p*‐value	Beta (S.E.)	*p*‐value	Beta (S.E.)	*p*‐value	Beta (S.E.)	*p*‐value
Fluid intelligence PGS	−0.001 (0.032)	0.981	0.047 (0.036)	0.193	−0.020 (0.034)	0.559	−0.037 (0.036)	0.303
Nonsexual assaultive trauma	−0.059 (0.038)	0.123	−0.089 (0.035)	0.013^*^	−0.004 (0.037)	0.906	0.062 (0.037)	0.087
Nonassaultive trauma	−0.055 (0.035)	0.115	−0.024 (0.034)	0.479	−0.028 (0.035)	0.412	0.026 (0.034)	0.456
Sexual assaultive trauma	0.092 (0.044)	0.035^*^	0.024 (0.048)	0.620	0.085 (0.036)	0.018^*^	0.067 (0.043)	0.117
**Fluid intelligence PGS × nonsexual assaultive trauma**	0.060 (0.034)	0.084	0.035 (0.037)	0.354	**0.093 (0.033)**	**0.006** ^**^	0.075 (0.036)	0.038^*^
Fluid intelligence PGS × nonassaultive trauma	0.041 (0.036)	0.710	−0.006 (0.039)	0.887	0.052 (0.034)	0.126	0.013 (0.034)	0.699
Fluid intelligence PGS × sexual assaultive trauma	−0.006 (0.036)	0.878	0.048 (0.043)	0.267	−0.053 (0.042)	0.213	0.011 (0.044)	0.793

*Note*: Sex, age, number of CPT sessions for each individual, principal components, and genotype array were included as covariates. All variables were modeled simultaneously. Significant results that survived a Bonferroni correction for eight tests (*p *< 0.00625) are bolded.

* *p *< 0.05.

** *p *< 0.01.

**FIGURE 1:**
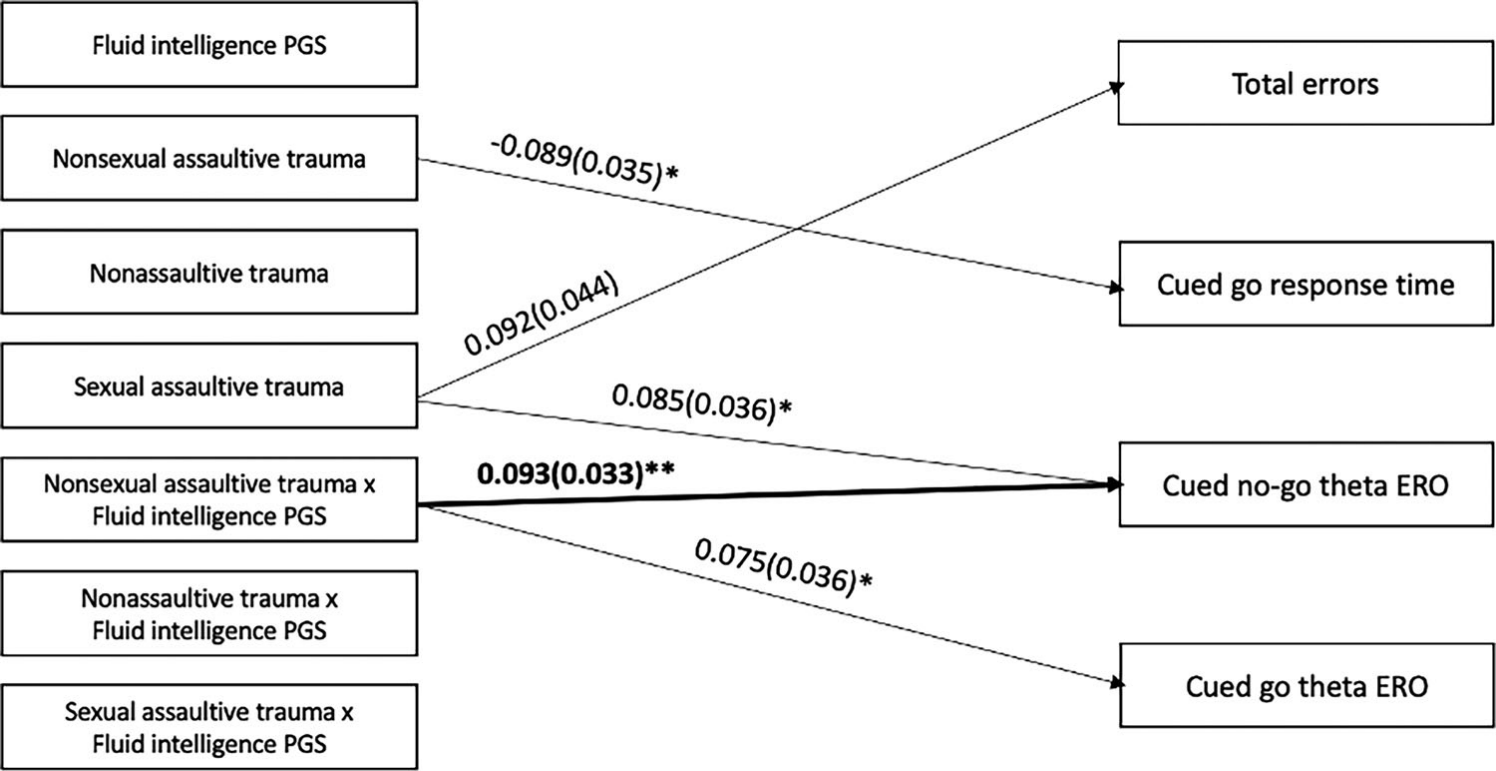
Associations of trauma, fluid intelligence polygenic score (PGS) and trauma × PGS interactions with performance and frontal theta event‐related oscillations (EROs) from the continuous performance test (CPT). *Note*: Only significant pathways are displayed. Significant results that survived a Bonferroni correction for eight tests (*p *< 0.00625) are bolded. Not pictured but are also included in this model are covariates: sex, age, number of CPT sessions for each individual, principal components, and genotype array. *p < 0.05; **p < 0.01.

**FIGURE 2:**
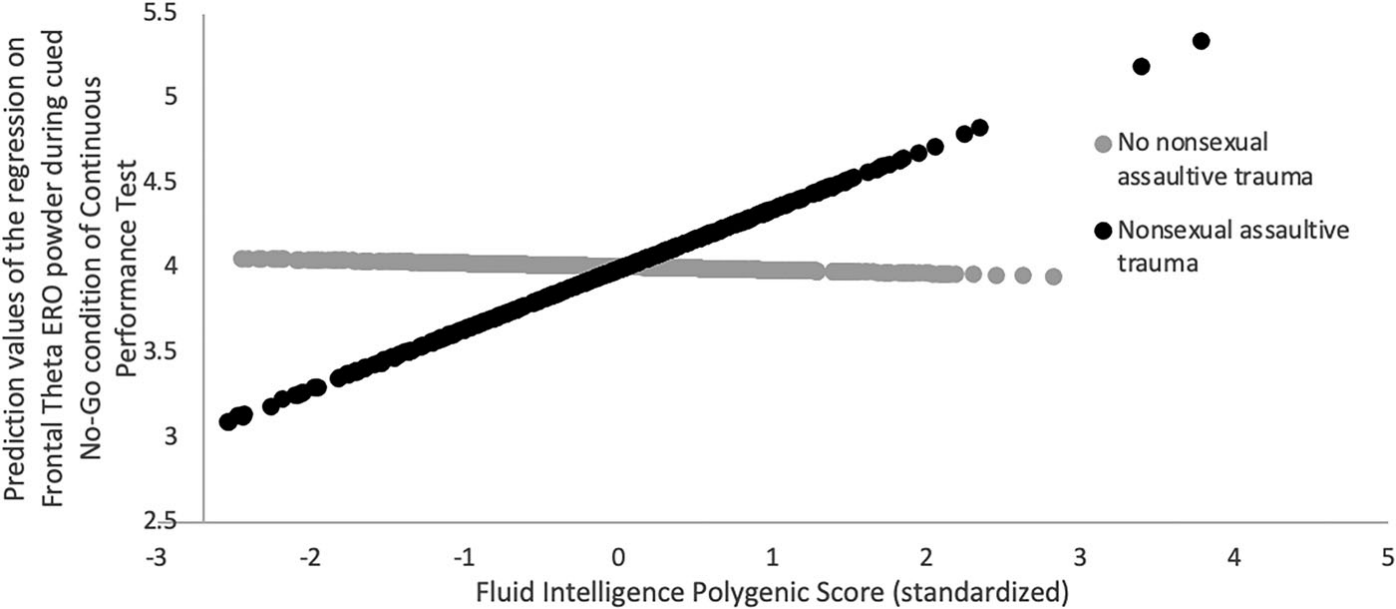
Interaction between fluid intelligence PGS and nonsexual assaultive trauma on frontal theta ERO total power during the cued no‐go condition of a continuous performance test.

Significant interaction effects between fluid intelligence PGS and nonsexual assaultive trauma were observed for cued no‐go and go theta ERO power such that individuals with lower fluid intelligence PGS and a nonsexual assaultive trauma had lower cued no‐go theta ERO power (*B* = 0.093, *p *< 0.01; Table 2) and lower cued go theta ERO power (*B* = 0.075, *p *< 0.05; Table 2). Only the interaction effect between the fluid intelligence PGS and nonsexual assaultive trauma on cued no‐go theta ERO power remained after a Bonferroni correction for eight tests (*p *< 0.00625; Table 2; Figure 1). A simple slopes plot visualizing this significant interaction is shown in Figure 2.

### 3.2 Exploratory Analyses Investigating Fluid Intelligence PGS × Nonsexual Assaultive Trauma × Sex on CPT Performance and ERO Measures

No significant interactions were observed for the fluid intelligence PGS × nonsexual assaultive trauma × sex interaction (Table 3). A significant main effect was observed for nonsexual assaultive trauma, such that individuals with a nonsexual assaultive trauma had shorter CPT cued go response times (*B* = −0.085, *p *< 0.05; Table 3). However, this did not survive a Bonferroni correction for eight tests (*p *< 0.00625). Significant main effects were observed for sex, such that female participants made less total CPT errors (*B* = −0.088, *p *< 0.05) and had longer CPT cued go response times (*B* = 0.113, *p *< 0.01; Table 3) compared to male participants. Only the main effect between sex and CPT cued go response time remained after a Bonferroni correction for eight tests (*p *< 0.00625).

**TABLE 3:** Three‐way interaction effects (fluid intelligence PGS × nonsexual assaultive trauma × sex) on continuous performance test (CPT) performance and frontal theta event‐related oscillations (EROs).

	Total errors		Cued go response time		Cued no‐go theta ERO		Cued go theta ERO	
Explanatory Variables	Beta (S.E.)	*p*‐value	Beta (S.E.)	*p*‐value	Beta (S.E.)	*p*‐value	Beta (S.E.)	*p*‐value
Fluid intelligence PGS	0.006 (0.033)	0.845	0.047 (0.037)	0.208	−0.018 (0.036)	0.624	−0.027 (0.037)	0.469
Nonsexual assaultive trauma	−0.062 (0.038)	0.109	−0.085 (0.036)	0.018^*^	−0.003 (0.037)	0.946	0.060 (0.037)	0.106
**Sex**	−0.088 (0.037)	0.018^*^	**0.113** **(0.035)**	**0.001** ^**^	0.012 (0.035)	0.732	−0.002 (0.036)	0.954
Fluid intelligence PGS × sex	−0.025 (0.036)	0.496	0.032 (0.037)	0.395	−0.005 (0.039)	0.897	0.032 (0.037)	0.389
Nonsexual assaultive trauma × sex	−0.021 (0.033)	0.521	0.046 (0.031)	0.140	0.028 (0.031)	0.363	−0.037 (0.032)	0.242
Fluid intelligence PGS × nonsexual assaultive trauma × sex	0.029 (0.032)	0.373	−0.019 (0.038)	0.626	0.009 (0.033)	0.794	0.030 (0.033)	0.363

*Note*: Nonassaultive trauma, sexual assaultive trauma, age, number of CPT sessions for each individual, principal components, and genotype array were included as covariates. All variables were modeled simultaneously. Significant results that survived a Bonferroni correction for eight tests (*p *< 0.00625) are bolded.

* *p *< 0.05.

** *p *< 0.01.

### 3.3 Main and Interaction Effects of PGS and Trauma on CPT Performance and ERO Measures

Significant main effects for nonsexual assaultive trauma and sexual assaultive trauma such that individuals with a nonsexual assaultive trauma had shorter CPT cued go response times (*B* = −0.089, *p *< 0.05; Table 2) and individuals with a sexual assaultive trauma had more total CPT errors (*B* = 0.092, *p *< 0.05; Table 2) and higher cued no‐go theta ERO power (*B* = 0.085, *p *< 0.05; Table 2). However, no significant main effects remained after a Bonferroni correction for eight tests (*p *< 0.00625; Table 2).

Significant interaction effects between the fluid intelligence PGS and nonsexual assaultive trauma were observed for cued no‐go and go theta ERO power such that individuals with lower fluid intelligence PGS and a nonsexual assaultive trauma had lower cued no‐go theta ERO power (*B* = 0.093, *p *< 0.01; Table 2) and lower cued go theta ERO power (*B* = 0.075, *p *< 0.05; Table 2). Only the interaction effect between the fluid intelligence PGS and nonsexual assaultive trauma on cued no‐go theta ERO power remained after a Bonferroni correction for eight tests (*p *< 0.00625; Table 2; Figure 1).

### 3.4 Exploratory Analyses Investigating Fluid Intelligence PGS × Nonsexual Assaultive Trauma × Sex on CPT Performance and ERO Measures

No significant interactions were observed for the fluid intelligence PGS × nonsexual assaultive trauma × sex interaction (Table 3). A significant main effect was observed for nonsexual assaultive trauma, such that individuals with a nonsexual assaultive trauma had shorter CPT cued go response times (*B* = −0.085, *p *< 0.05; Table 3). However, this did not survive a Bonferroni correction for eight tests (*p *< 0.00625). Significant main effects were observed for sex, such that female participants made less total CPT errors (*B* = −0.088, *p *< 0.05) and had longer CPT cued go response times (*B* = 0.113, *p *< 0.01; Table 3) compared to male participants. Only the main effect between sex and CPT cued go response time remained after a Bonferroni correction for eight tests (*p *< 0.00625).

## 4 Discussion

This study investigated interaction effects between cognitive function PGS and types of trauma exposure on CPT performance and frontal theta EROs. A significant interaction effect between the fluid intelligence PGS and nonsexual assaultive trauma was observed. Individuals with lower fluid intelligence PGS and a nonsexual assaultive trauma had lower frontal theta ERO power during the no‐go condition of the CPT compared to individuals with higher fluid intelligence PGS. These results suggest nonsexual assaultive trauma may moderate associations between polygenic factors and neural function.

To our knowledge, no study has examined how trauma moderates the relationship between PGS of cognitive function on neural response inhibition. The most significant finding involved interaction effects between the fluid intelligence PGS and nonsexual assaultive trauma on frontal theta ERO power during the cued no‐go condition of the CPT. Fluid intelligence is a higher‐level cognitive skill that represents the ability to reason in novel situations (Aydmune et al. 2020). Inhibition is an executive function associated with the prefrontal cortex, involving cognitive processes related to control of emotions, thoughts, and conduct during goal‐directed behavior (Aydmune et al. 2020), and has been shown to play a role in fluid intelligence (Aydmune et al. 2020; Diamond 2013; Michel and Anderson 2009). Nonsexual assaultive trauma exposure negatively affects inhibition development, leading to adverse consequences such as substance problems and aggressive behavior (Bounoua et al. 2020). While inhibition contributes to fluid intelligence development and trauma exposure can negatively affect inhibition, and the literature suggests a genetic component to cognitive function, no study has investigated how trauma influences the relationship between genetic risk for cognitive impairment and cognitive processing later in life.

Lower frontal theta EROs during no‐go conditions indicate less efficient neural synchronization, observed in individuals with AUD or with a family history of AUD, and those exposed to childhood sexual assaultive trauma (Meyers et al. 2019; Pandey et al. 2016). This study demonstrated that nonsexual assaultive trauma combined with lower fluid intelligence PGS makes individuals more likely to present with lower frontal theta ERO power during the cued no‐go portion of the CPT. Lower frontal theta power during the cued no‐go indicates less efficient neural synchronization during behavioral inhibition in those exposed to nonsexual assaultive trauma and lower fluid intelligence PGS. This suggests nonsexual assaultive trauma, but not nonassaultive or sexual assaultive, interacts with polygenic scores for fluid intelligence, resulting in neural differences that are seen as correlates of cognitive function. This is in line with previous research that has shown individuals exposed to assaultive trauma have worse outcomes regarding cognitive function and psychopathology, as compared to those exposed to nonassaultive trauma (Cisler et al. 2011; Meyers et al. 2019). While neural differences were observed (i.e., lower frontal theta ERO power), no significant behavioral differences were observed. This is consistent with studies where participants show similar behavioral performance with differences in neural activity (Camerer and Mobbs 2017; Yu et al. 2025). These studies suggest this may be due to neural flexibility and indicate that different individuals may use different brain mechanisms to perform the same task.

While previous work has also implicated adverse effects of sexual assaultive trauma, fewer individuals in the analytic sample were exposed to sexual assaultive trauma compared to other trauma, potentially limiting the power to detect these complex interactive effects. While PGS typically explain only a small percentage of the variance in a trait (Bogdan et al. 2018), future studies can improve these analyses by increasing sample sizes for all groups. There were no significant effects fluid intelligence PGS or nonsexual assaultive trauma alone; only in the interaction did frontal theta ERO differ for the cued no‐go condition. This suggests that genetic risk for fluid intelligence and exposure to nonsexual assaultive trauma be present together to cause the ERO difference. Future studies should consider trauma type, particularly nonsexual assaultive trauma, when investigating neural function risk. Future research could use these altered frontal theta EROs as potential neural markers to predict outcomes like AUD, PTSD, or cognitive outcomes, like fluid intelligence scores, later in life. Understanding how genetic and environmental influences affect neural function and cognitive function is necessary to identify individuals who may benefit from targeted early intervention to prevent severe consequences of these genetic risks and traumatic exposures.

Exploratory analyses were conducted to investigate sex differences in any significant interaction findings. There were no significant findings from the three‐way interaction (fluid intelligence PGS × nonsexual assaultive trauma × sex), but a main effect of sex showed female participants had longer response times and fewer errors, while male participants had shorter response times and more errors. This suggests an impairment in response inhibition in men, but not women. These findings are consistent with the literature that suggests women have greater inhibitory control than men (Mansouri et al. 2016; Sjoberg and Cole 2018). Enhanced inhibitory control has been associated with avoidance symptoms in disorders, such as generalized anxiety disorder (Grillon et al. 2017). Further, women are more likely to be diagnosed with these disorders (Bandelow and Michaelis 2015). However, impaired inhibitory control has been associated with impulsive behavior and addiction, which are observed more in males (Mansouri et al. 2016). The results from the current study and from previous research demonstrate the importance of considering sex differences when investigating response inhibition.

### 4.1 Limitations

This study was only conducted in individuals of European‐like ancestry. Future studies should expand on these findings in ancestrally diverse populations.

We did not account for psychiatric disorders that are often comorbid with trauma exposure and have been shown to influence neural and cognitive function. Future studies should investigate how trauma type affects these disorders as well as any interaction effects on neural or cognitive outcomes, especially in a sample such as COGA's prospective study.

The SSAGA interview does not ask about recurrent trauma exposure. Therefore, this was not accounted for in our study, which may bias findings, since studies have shown that individuals exposed to one trauma are more likely to have recurrent trauma exposure (Strom et al. 2020). Recurrent trauma exposure may influence the severity of neural and cognitive differences, which also could not be captured given the limitation of the data. Future studies would benefit by collecting information on recurring trauma exposure for each type of trauma.

This study did not investigate the influence of the age of first trauma exposure. This was excluded to primarily investigate how individual trauma types moderate associations between polygenic scores of fluid intelligence and neural and cognitive function. Future studies should include these to determine if the timing of trauma affects our findings, especially if trauma is first experienced during critical developmental periods.

This study was a cross‐sectional analysis, utilizing data from the last session available for each individual. Future studies should consider a longitudinal analysis to observe how EROs during response inhibition may change over time after a traumatic exposure and identify a developmental period when trauma exposure may affect these neural changes most.

## 5 Conclusion

Our study showed that individuals exposed to nonsexual assaultive trauma with lower fluid intelligence PGS had lower frontal theta ERO power during the cued no‐go condition of the CPT, indicating less efficient neural synchronization during behavioral inhibition. These findings suggest that nonsexual assaultive trauma interacts with polygenic scores for fluid intelligence, resulting in altered neural function in adulthood. The main effect observed for sex suggests female participants have better inhibitory control as compared to men. Future studies should consider trauma type when investigating associations between genetic vulnerability to complex cognitive functions, including response inhibition, via polygenic scores. Understanding how trauma influences cognitive outcomes for individuals with genetic risk can inform early intervention and treatment strategies for individuals at high risk for trauma exposures and altered neural function.

## Author Contributions

**Stacey Saenz de Viteri**: conceptualization, methodology, investigation, formal analysis, writing – original draft, writing – review and editing. **Ashwini Pandey**: data curation, writing – review and editing. **Chella Kamarajan**: writing – review and editing. **Gayathri Pandey**: writing – review and editing. **Weipeng Kuang**: writing – review and editing. **Sivan Kinreich**: writing – review and editing. **Grace Chan**: writing – review and editing. **Martin H. Plawecki**: writing – review and editing. **Marc A. Schuckit**: writing – review and editing. **Bernice Porjesz**: conceptualization, supervision, funding acquisition, resources, writing – review and editing. **Jacquelyn L. Meyers**: conceptualization, supervision, funding acquisition, resources, writing – review and editing.

## Conflicts of Interest

There are no conflicts of interest to disclose.

## Peer Review

The peer review history for this article is available at https://publons.com/publon/10.1002/brb3.70729.

## Supporting information




**Supporting Material**: brb370729‐sup‐0001‐SuppMat.docx

## Data Availability

The data that support the findings of this study are available from the corresponding author upon reasonable request.
