# Supplementary material for: Frontal Theta Event‐Related Oscillations During a Continuous Performance Test: The Influence of Trauma Type and Fluid Intelligence Polygenic Score
Source: Brain Behav. 2025 Aug 4;15(8):e70729. doi: 10.1002/brb3.70729 (PMC12321974; doi:10.1002/brb3.70729)
Supplement: Supplementary file 1 — Supporting Material: brb370729‐sup‐0001‐SuppMat.docx [file BRB3-15-e70729-s001.docx]

**Supplementary Material**

**ADDITIONAL MATERIALS AND METHODS**

*Event-related oscillations recording and processing*

Participants were comfortably seated in a dimly lit, sound-attenuated, electrically-shielded room 1 m away from a computer monitor screen (IAC Acoustics, Bronx, NY). The EEG was recorded on a Neuroscan System (versions 4.1-4.5; Neurosoft, Inc. El Paso, TX) using a 61-channel electrode cap (Electro-cap International, Inc., Eaton, OH) that had electrode placements consistent with the extended 10-20 International System (American Clinical Neurophysiology Society, 1991). Reference electrodes were located on the tip of the nose, and grounding electrodes were placed on the forehead. Time-locked EEG activity to both cued Go (response activation; “X” preceded by “O”) and cued No-Go (response inhibition; “O” followed by a distractor letter “A-L”) were recorded at frontal, central and parietal loci, and event-related oscillation (ERO) characteristics are measured. As described in previously published studies from our laboratory (Pandey et al., 2016), all recordings were digitally re-sampled offline at 256 samples per second with 0.3-45 Hz band pass filter in order to control for the DC drifts and AC power artifacts in the waveform. The EEG segments were divided into epochs of 1,625 ms. All epochs exceeding +/- 100 V amplitude were excluded from further processing. Each participant’s successful trials were averaged based on task conditions and inspected to eliminate trials that show any kind of artifact. S-transformation was used to obtain estimates localized power of non-stationary evoked potential time series (Stockwell, 2007). Our laboratory has previously implemented this method to evaluate event-related signals in the time-frequency domain in COGA’s prospective study (Kamarajan et al., 2006; Meyers et al., 2019; Pandey et al., 2016).
